# Does social capital travel? Influences on the life satisfaction of young people living in England and Spain

**DOI:** 10.1186/1471-2458-12-138

**Published:** 2012-02-21

**Authors:** Antony R Morgan, Francisco Rivera, Carmen Moreno, Bo JA Haglund

**Affiliations:** 1 Public Health Sciences Department, Karolinska Institute, Stockholm, Sweden; 2 Department of Clinical, Experimental and Social Psychology, University of Huelva, Huelva, Spain; 3 Department of Developmental and Education Psychology, University of Seville, Seville, Spain

## Abstract

**Background:**

This study used a social capital framework to examine the relationship between a set of potential protective ('health assets') factors and the wellbeing of 15 year adolescents living in Spain and England. The overall purpose of the study was to compare the consistency of these relationships between countries and to investigate their respective relative importance.

**Methods:**

Data were drawn from the 2002, English and Spanish components of the WHO Health Behaviour in School-Aged Children (HBSC) survey A total of 3,591 respondents (1884, Spain; 1707, England) aged 15, drawn from random samples of students in 215 and 80 schools respectively were included in the study. A series of univariate, bivariate and multivariate (general linear modelling and decision tree) analyses were used to establish the relationships.

**Results:**

Results showed that the wellbeing of Spanish and English adolescents is similar and good. Three measures of social capital and 2 measures of social support were found to be important factors in the general linear model. Namely, family autonomy and control; family and school sense of belonging; and social support at home and school. However, there were differences in how the sub components of social capital manifest themselves in each country--feelings of autonomy of control, were more important in England and social support factors in Spain.

**Conclusions:**

There is some evidence to suggest that social capital (and its related concept of social support) do travel and are applicable to young people living in Spain and England. Given the different constellation of assets found in each country, it is not possible to define exactly the precise formula for applying social capital across cultures. This should more appropriately be defined at the programme planning stage.

## Background

This study used a social capital framework to examine the relationship between a set of potential protective ('health assets') factors and wellbeing amongst 15 year adolescents living in Spain and England The overall purpose was to compare the consistency of these relationships between countries and to investigate their respective relative importance. In addition it explored whether there was an optimum profile of factors that maximised the possibilities of wellbeing in the study population.

### The concept of wellbeing

Wellbeing was the chosen outcome of interest as it is increasingly seen as an important component of broader strategies designed to improve outcomes of children and young people [1,2] and an evidence base exists [3] to suggest that it provides a pathway to health. However, like other health related concepts, a lack of precise definition [4,5] with complicated amalgamations of ideas can make it difficult to understand how research can be translated into practicable actions. For example, UNICEF's [6] study of child wellbeing in rich countries uses a large composite indicator, amassing a whole range of inputs and outputs related to health. The league table produced presents Spanish adolescents near the top and their English counterparts lagging behind at the bottom, but provides no insights into how improvements could be made.

Other definitions distinguish between emotional, psychological and social wellbeing, defining the totality as 'a positive state of mind and body, feeling safe and able to cope, with a sense of connection with people, communities and the wider environment' [7]. Aspects of this definition encompass notions of social capital. Deciding on the most appropriate definition in some ways depends on the purpose upon which it is being used. The more complex, may be appropriate for assessing global scores of wellbeing between or within countries. However, if the aim is to understand the mechanisms through which it is produced, then more specific representations need to be employed.

The study presented here, highlights the potential for social capital to support the production of wellbeing as an intermediate outcome along the pathway to health. Wellbeing is represented by the singular measure of life satisfaction as it provides a useful proxy and an immediately obvious benchmark for those responsible for thinking about young people's health programmes.

### Social capital and young people

Most of the research conducted over the last 10 years on the usefulness of social capital as a health related concept has focused on adult health (examples include: [8-12]). Disciplinary territorial wars and debating points aside, this literature points towards social capital or at least its underlying constructs (social relationships, levels of trust, group membership and civic engagement) as being beneficial for health across different ethnic groups, generations and gender [13]. However, the exact relationship between these constructs and different outcomes vary [14] and some authors suggest that after socio-economic status is taken into account, the predictive value of social capital is considerably weakened [15,16]. Nonetheless used as an adjunct to other strategies to tackle the structural determinants of health, it is generally deemed to be beneficial [17,18].

**Figure 1:**
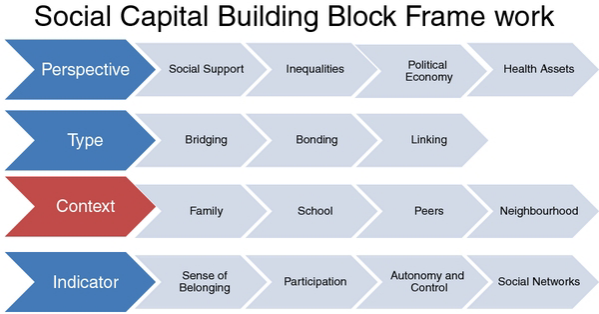
**Social Capital building block framework**.

Social capital research as it relate to young people's health was later to develop but in the main has followed a similar pattern to the adult literature, showing some benefits for health [19-22]. Other HBSC studies have shown specific links between social capital and life satisfaction [23]. That said similar to the adult evidence base, a lack of consistency over definition and measurement issues makes the synthesis of the available evidence difficult. Morgan [24] calls for a more systematic evidence base and has put a forward a 4 stage building block framework (perspectives, type of social capital, context and indicators) to help guide future research--see Figure 1.

The present study focuses on two of those building blocks namely perspectives and context.

### Perspective

Previously Szreter and Woolcock [25] have argued that social capital needs to be properly understood if it is to make a significant contribution to public health theory and highlight the three perspectives, that if made explicit may help clarify why and how social capital can be applied in practice. The perspectives are: social support (the ability to draw on resources through connections to others), inequalities (the erosion of citizen's sense of social justice and inclusion), and political economy (exclusion from material resources). Kawachi et al. [26] found these accounts useful to help unpack and distinguish between the different forms of social capital, bridging, bonding and linking [27,28] providing a better chance of understanding the precise mechanisms through which social capital can facilitate a range of health related outcomes. Morgan [24] adds another perspective--'health assets' argued to be helpful in thinking about the concept's relevance to young people. The asset approach has emerged recently [29,30] as one way in which policy makers, researchers and practitioners, can think differently about maximising opportunities for health, by identifying the capacities and capabilities of individuals and communities rather than focusing on problems and failures. The idea brings a number of well established ways of working with young people to the fore. Firstly, health might more easily be achieved if they are seen as 'social agents' active in the construction and determination of their own social lives, and the lives of those around them and of the societies in which they live rather than passive recipients of health programmes [31,32]. Secondly, existing research [33,34] on positive youth development highlights that the more young people are provided with opportunities to experience and accumulate those factors ('health assets') known to be protective of health, the more likely they are to achieve positive outcomes. The Search Institute http://www.search-institute.org/ has identified 40 development assets, spanning, family dynamics, support from community adults, school effectiveness, peer influence, values development, and a range of specific skills and competencies required for young people to thrive. The health asset approach seeks to understand whether some assets are more important than others; the benefit of accruing them and their stability across different social and cultural contexts.

Morgan (2010) observed many of these development assets mirrored the core constructs of social capital and as such identified it as a potential health asset. Placing social capital in this context helps to ward off past criticism of the 'downside' of social capital [35] as by definition, it sets it as a resource for 'public good'. Whereas, strong inward looking networks that result in the exclusion of individuals or parts of communities would not be seen as a protective factor in an asset lexicon. It also answers the call by Szreter and Woolcock [25] to make explicit social capital's purpose and illustrates that the different disciplinary perspectives need not be in opposition. For example, the asset approach proposes, that earlier on in life individual accounts of social capital (for example Bourdieu [36]) are appropriate as young people need to accumulate a set of social resources that enable access to, and participation in a range of different types of networks to improve their opportunities for wellbeing and health. In addition, given one of its core principles is to seek active youth participation, building feelings of autonomy and control raises young people's confidence and willingness to participate in a range of informal and formal networks [37]. This may lead to enhanced participation in community life conducive to Putnam's [38] collective notion of social capital.

### Context

The multi-component nature of social capital has often been argued to be its weakness, some questioning whether by incorporating so many disparate social phenomena into one concept, loses its distinct meaning [39]. This paper proposes that it is its complexity that gives a strength over other concepts. However, only if research can help to unravel its individual constructs. Outlining, linking and explaining them as pathways to health and related outcomes. Part of this unravelling requires an examination of social capital in context, recognising that its exact configuration may vary across gender, age and culture [13]. For example, Morrow [40] found that young people put greater importance on their interpersonal networks based on friendship and family to secure their sense of belonging and well-being rather than being members of formal community networks and associations and that school was an important 'community' in its own right.

This study contributes to a better understanding of context by assessing the relative importance of young people's contexts by assessing the relative importance of young people's social capital in the home, at school, in the neighbourhood and amongst peers. The study also tests how robust the relationships are in 2 different country contexts.

Table 1 summarises the social capital framework. A number of social support indicators are also included as it is recognised that although a conceptually different concept, it offers one positive exchange that might arise from involvement in social networks. For the purposes of this analysis, the indicators will be referred to as potential candidate assets.

**Table 1:** Social capital framework: summary of independent variables

Context	Concept	Construct	Items
Family	Social Capital	Family sense of belonging (FSB)	Family doing things together (every day; once a week; less often; never). Watch TV or video; play indoor games; eat meals; go for a walk; going places together; visiting friends or relatives; play sports; sitting and talking
		Autonomy and Control (FAC)	Mother/father--asked separately (almost always, sometimes, never): Let me do the things I like doing, like me to make my own decisions, try to control everything I do, treat me like a baby.
	Social Support	Family social support (FSS)	Mother/father ((asked separately): helps me as much as I need; is loving; understands my problems and worries; makes me feel better when I am upset)

School	Social capital	School sense of belonging (SSB)	The students in my class enjoy being together; other students accept as I am; students are kind and helpful (strongly agree to strongly disagree) Our teachers treat us fairly; I am encouraged to express my own views in class. (strongly agree through to strongly disagree):
	Social support	Autonomy and control (SAC) School social support (SSS)	Most of my teachers are friendly; my teachers are interested in me as a person; when I need extra help I can get it(strongly agree through strongly disagree):

Neighbourhood	Social capital	Neighbourhood sense of belonging (NSB)	People stop to talk to one another in the street; it is safe for young people to play outside during the day; you can trust people round here; I could ask for help or favour from a neighbour; most people around here would take advantage of you if they got the chance; there are good places to spend your time(strongly agree through strongly disagree)

Peers	Social support	Communication with friends (PSS)	How easy is it for you to talk to your friends about things that really bother you?. Asked for same sex friends and opposite sex friends separately(every easy through very difficult):

## Methods

### Study population and design

Data were drawn from the 2002, English and Spanish components of the WHO Health Behaviour in School-Aged Children (HBSC) survey [41], an international study on the health and related behaviour of 11, 13 and 15 year olds in their social context. This study used data from 15 year olds, as previous HBSC analysis has shown wellbeing to decline with increasing age and the determinants of health at this key development stage to be distinct. A total of 3,591 respondents (1884, Spain; 1707, England), drawn from random samples of students in 215 and 80 schools respectively were included in the study. The overall achieved student response rate was 82% in Spain and 76% in England. Data was collected by a standardised questionnaire under supervised conditions [42]. Questions relating to social capital were obtained from optional packages selected by countries to supplement their core questionnaire. Further details of the Spanish and English methods can be found in Moreno et al. [43] and Morgan et al. [44].

### Outcome

Life satisfaction was measured by the well validated Cantril Ladder [45]. It is included in the HBSC study, as it represents an important cognitive aspect of the complex, multi-faceted concept of wellbeing [46]. Respondents were presented with the picture of a ladder and asked to choose a position on one of the 10 steps (top of the ladder represented the best possible life and 0 the worst) to indicate how they felt about their life at the moment.

### Independent variables

Proper conceptualisation of social capital relies on good measurement, which some have argued has hampered its development [14,47]. The social capital framework used here was adapted from Morrow's [48] original qualitative work exploring the concepts relevance to young people. Two out of three sub domains identified: sense of belonging (identity and safety with their environments) and autonomy and control (perceptions of power to influence decisions) were used in the analysis. The third, social networking (participation in school and community life) could not be included as this was omitted from the Spanish questionnaire. A total of five indicators of social capital were included across each of the contexts and 3 indicators of social support. Table 1 describes the items used to construct a set of composite indicators which represent the candidate assets identified as potentially protective of young people's wellbeing. A 3 level variable was created: low, medium, high and the high category used as the reference category. These categories were derived by scoring each of the responses, summing the scores and assigning the results to each of the categories. For example, the candidate asset 'school sense of belong' is made up of 3 statements: the students in my class enjoy being together; students in my class are kind and helpful and; other students accept me as I am. There were 5 possible response categories and these were scored as follows: strongly agree (5); agree (4); neither agree and disagree(3); disagree (2); strongly disagree (1). The scores ranged from 3-15 and for the purpose of the analysis were grouped as: low SSB(3.0-7.5); medium SSB (7.6-11.9) and high SSB (12.0-15.0).

### Socio-demographic factors

The role of gender and socio-economic status (as represented by the family affluence scale) (FAS) were also considered in the analysis.

#### FAS

FAS has been developed by the HBSC study to approximate for socio-economic status given the known difficulties of asking young people to detail accurately their parents occupation (see Currie et al. [49] for further details). Four items contribute to this summary measure: does your family own a car; do you have your own bedroom to yourself; during the past 12 months how many times did you travel away on holiday; how many computers does your family own. A 3 level score was created low, medium and high FAS.

### Data analysis

The study objectives were achieved through a series of univariate, bivariate and multivariate analyses. Four steps were taken to compare the influence of social capital on life satisfaction in the 2 countries. Given the size of the sample, tests of significance (to test the null hypothesis) and effect size (to assess the magnitude of difference) were carried out at each stage. Levels of significance were set at *p *< 0.001.

The univariate analysis established the general distribution of the candidate assets (Table 2), using the X^2 ^statistic for significance and Phi coefficients and Cramer's *V *for effect size (their respective use dictated by type of variable). Effect size ranges were: 0-0.09, negligible effect; 0.10-0.29, low; 0.30-0.49, medium, and > 0.50, large effect [50]. The bivariate analysis (Table 3) examined associations between independent and dependent variables using the X^2 ^statistic, student's t and ANOVA for significance and Cohen's *d *for effect size. Effect size statistics classified as: 0-0.19, negligible; 0 20-0.49, low; 0.50-0.79, medium, > 0.80, large [51].

**Table 2:** Univariate Analysis Independent Variables by country

		Spain	England	**X**^***2***^	*df*	**sig**.	Phi/Cramer's *V*
				
		%	%				
**Gender**	Boy	47%	46%	**0.541**	**1**	**0.462**	**0.012**
					
	Girl	53%	54%				

**Family affluence scale (FAS)**	Low	28%	7%	**287.48**	**2**	**< 0,001**	**0.282**
					
	Middle	48%	66%				
					
	High	24%	27%				

**Family sense of belonging (FSB)**	Low	29%	30%	**6.217**	**2**	**0.045**	**0.042**
					
	Middle	43%	45%				
					
	High	28%	25%				

**Family autonomy and control (FAC)**	Low	10%	3%	**194.044**	**2**	**< 0,001**	**0.244**
					
	Middle	44%	28%				
					
	High	46%	69%				

**Family social support (FSS)**	Low	7%	12%	**37.324**	**2**	**< 0,001**	**0.108**
					
	Middle	19%	22%				
					
	High	74%	66%				

**School sense of belonging (SSB)**	Low	5%	10%	**130.558**	**2**	**< 0,001**	**0.190**
					
	Middle	40%	53%				
					
	High	55%	37%				

**School autonomy and control (SAC)**	Low	8%	10%	**7.244**	**2**	**0.027**	**0.045**
					
	Middle	55%	52%				
					
	High	37%	38%				

**School social support (SSS)**	Low	7%	6%	**3.296**	**2**	**0.192**	**0.030**
					
	Middle	61%	59%				
					
	High	33%	35%				

**Neighbourhood sense of belonging (NSB)**	Low	13%	24%	**93.212**	**2**	**< 0,001**	**0.168**
					
	Middle	66%	63%				
					
	High	21%	12%				

**Peers social support (PSS)**	Low	10%	7%	**13.83**	**2**	**0.001**	**0.063**
					
	Middle	28%	25%				
					
	High	62%	68%				

**Table 3:** Bivariate analysis: associations between social capital and related factors on life satisfaction of adolescents, Spain and England

		Spain			England			Spain	England	Spain		England	
		**Mean**	**Std. Dev**.	**Recount**	**Mean**	**Std. Dev**.	**Recount**	**Significant test**		**Cohen's d**			

**Total Mean Life Satisfaction**		**7.15**	**1.66**	**1,884**	**7.00**	**1.82**	**1,707**	*t = 2.533*		*0.09*			

Gender	Boy	7.30	1.50	864	7.15	1.74	833	*t *= 3,775	*t *= 3,077	B-G	0.18	B-G	0.15
				
	Girl	7.01	1.75	972	6.88	1.87	984	< 0.001	0.002				

FAS	Low	6.96	1.74	507	6.21	1.91	119	*F *= 4,586	*F *= 29,970	L-M	-0.15	L-M	-0.38
				
	Middle	7.20	1.56	879	6.90	1.84	1,185			M-H	-0.04	M-H	-0.31
	
	High	7.26	1.66	437	7.44	1.62	480	0.01	< 0.001	L-H	-0.17	L-H	-0.74

FSB	Low	6.57	1.88	515	6.50	1.95	535	*F *= 51,521	*F *= 34,662	L-M	-0.43	L-M	-0.34
				
	Middle	7.27	1.42	767	7.11	1.68	789			M-H	-0.19	M-H	-0.19
	
	High	7.55	1.50	508	7.44	1.72	434	< 0.001	< 0.001	L-H	-0.58	L-H	-0.51

FAC	Low	6.23	2.04	163	5.12	2.42	49	*F *= 47,641	*F *= 72,545	L-M	-0.52	L-M	-0.73
				
	Middle	7.09	1.54	734	6.48	1.81	443			M-H	-0.28	M-H	-0.53
	
	High	7.51	1.45	762	7.38	1.65	1,102	< 0.001	< 0.001	L-H	-0.82	L-H	-1.34

FSS	Low	5.87	2.23	107	5.94	2.20	192	*F *= 76,706	*F *= 68,595	L-M	-0.44	L-M	-0.39
				
	Middle	6.66	1.63	316	6.67	1.68	350			M-H	-0.55	M-H	-0.45
	
	High	7.45	1.40	1,198	7.42	1.63	1,034	< 0.001	< 0.001	L-H	-1.07	L-H	-0.85

SSB	Low	5.99	2.33	88	5.95	2.29	176	*F *= 43,077	*F *= 40,836	L-M	-0.56	L-M	-0.55
				
	Middle	6.91	1.54	724	6.96	1.73	958			M-H	-0.32	M-H	-0.22
	
	High	7.41	1.58	1,000	7.34	1.68	662	< 0.001	< 0.001	L-H	-0.86	L-H	-0.76

SACl	Low	6.63	1.93	135	6.33	2.33	175	*F *= 23,817	*F *= 30,413	L-M	-0.23	L-M	-0.27
				
	Medium	7.01	1.62	991	6.84	1.82	931			M-H	-0.29	M-H	-0.32
	
	High	7.47	1.56	674	7.38	1.56	691	< 0.001	< 0.001	L-H	-0.52	L-H	-0.60

SSS	Low	6.51	1.97	117	6.13	2.33	100	*F *= 36,648	*F *= 29,689	L-M	-0.28	L-M	-0.38
				
	Middle	6.98	1.64	1,082	6.83	1.82	1,063			M-H	-0.39	M-H	-0.32
	
	High	7.60	1.47	580	7.40	1.62	627	< 0.001	< 0.001	L-H	-0.70	L-H	-0.74

NSB	Low	6.63	1.76	206	6.50	2.03	427	*F *= 19,801	*F *= 25,860	L-M	-0.30	L-M	-0.34
				
	Middle	7.10	1.52	1,022	7.11	1.70	1,112			M-H	-0.27	M-H	-0.22
	
	High	7.51	1.63	332	7.48	1.68	218	< 0.001	< 0.001	L-H	-0.53	L-H	-0.51

PSS	Low	6.82	1.81	172	6.55	1.89	130	*F *= 5,805	*F *= 4,714	L-M	-0.14	L-M	-0.30
				
	Middle	7.04	1.57	499	7.09	1.81	432			M-H	-0.12	M-H	0.02
	
	High	7.24	1.63	1,102	7.05	1.78	1,189	0.03	0.009	L-H	-0.25	L-H	-0.28

The general linear model (GLM) assessed how much of the dependent variable was explained by the candidate assets independently (Table 4). Standardised coefficients of regression and the partial square eta statistic were used to summarise the weight of explanation that could be accounted for by each factor. Effect size classification: 0-0.009 negligible; 0.010-0.089 small; 0.090-0.249, medium; and > 250, large effect [51].

**Table 4:** General Linear Model for Spain and England

	Spain					England				
	**Sum of Squares**	***df***	***F***	**Sig**.	**Partial Eta Squared**	**Sum of Squares**	***df***	***F***	**sig**.	**Partial Eta Squared**

Corrected Model	617.03	19	16.58	< 0,001	**0.196**	936.55	19	19.43	< 0,001	**0.207**

Intercept	6736.00	1	3439.96	< 0,001	**0.727**	5538.96	1	2183.04	< 0,001	**0.607**

**FSB**	18.11	2	4.62	0.01	0.007	38.44	2	7.57	< 0,001	**0.011**

**FAC**	33.91	2	8.66	< 0,001	**0.013**	98.02	2	19.32	< 0,001	**0.027**

**FSS**	79.99	2	20.43	< 0,001	**0.031**	67.37	2	13.28	< 0,001	**0.018**

**SSB**	71.77	2	18.33	< 0,001	**0.028**	61.33	2	12.09	< 0,001	**0.017**

SAC	0.44	2	0.11	0.89	0.000	13.39	2	2.64	0.07	0.004

**SSS**	31.97	2	8.16	< 0,001	**0.012**	7.46	2	1.47	0.23	0.002

NSB	13.20	2	3.37	0.03	0.005	20.66	2	4.07	0.02	0.006

PSS	10.62	2	2.71	0.07	0.004	14.48	2	2.85	0.06	0.004

**FAS**	4.96	2	1.27	0.28	0.002	55.83	2	11.00	< 0,001	**0.015**

Gender	15.17	1	7.75	0.01	0.006	18.78	1	7.40	0.01	0.005

Error	2529.94	1,292	1.95816			3580.09	1,411	2.537271		

Total	70,724	1,312				76,510	1,431			

	R Squared = ,196 (Adjusted R Squared = ,184)					R Squared = ,207 (Adjusted R Squared = ,197)				

**Figure 2:**
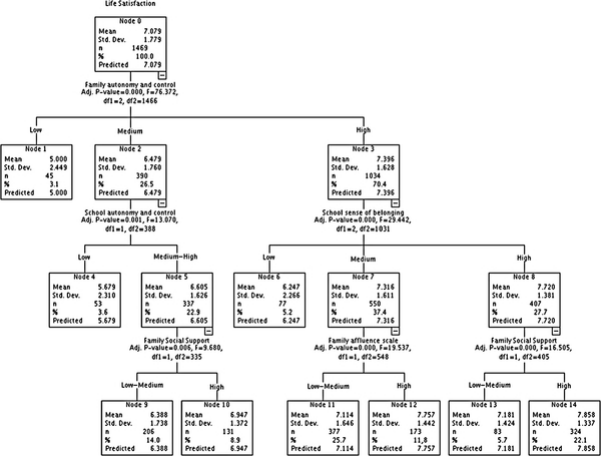
**Tree analysis England**.

**Figure 3:**
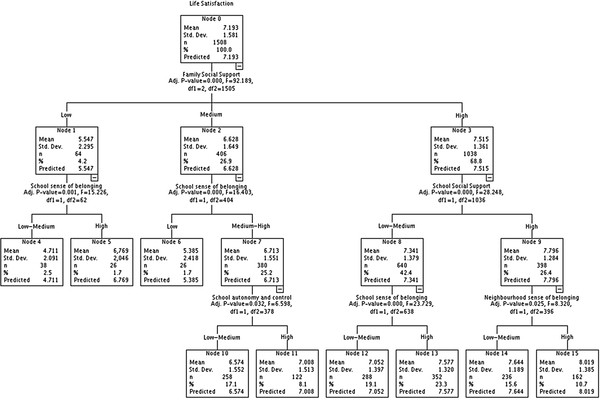
**Tree analysis Spain**.

Decision tree analysis (Figures 2 and 3) was used to determine how best the significant candidate assets combined to predict the variable [52]. This study used the exhaustive Chi-squared Automatic Interactive Detector (CHAID) algorithm to select a set of predictors and their interactions that optimally predict life satisfaction. Each candidate asset was assessed to see if splitting the sample based on this predictor led to a statistically significant discrimination in life satisfaction. Node 0 of the decision tree identifies the most important factor and new branches of the 'tree' emerge as the analysis develops an algorithm (represented by consecutively numbered nodes) highlighting the order of importance of each candidate asset. The tree continues to divide until no further significant discrimination of variables can be found. The F statistical test (ANOVA) assesses the statistical significance of each of the segmentations. It provides a clear representation of the potential candidate assets, highlighting the optimum configuration that may maximize life satisfaction.

SPSS version 17.0 was used for all the analysis which includes the 'software answer tree' package.

The HBSC network requires all countries to follow the ethical requirements set by their national or regional ethical authority and details of this are kept in the international databank in Bergen, Norway. This study is a secondary analysis of the data collected in England and Spain and therefore does not require ethical clearance. However the original surveys upon which the study is based: gained ethical approval from the University of Seville Ethics Committee in Spain. The English survey was carried out by the British Market Research Bureau, who follow the Market Research Society (MRS) code of conduct and data protection to maintain their ethical standards. Both countries adopted a passive student and parent opt out scheme.

## Results

Overall there were no statistical differences in life satisfaction between Spanish and English adolescents (means of 7.15 and 6.99 respectively) (t = 2.533, *p *> 0.01, Cohen's *d *0.09)

### Univariate analysis

Table 1 shows the frequency distributions of all independent variables of interest for Spain and England. No statistical differences in the patterns of distribution between the 2 countries were found for gender, sense of family belonging (SSB), school autonomy and control (SAC), school social support (SSS) or peer social support (PSS). Differences were observed however, for family autonomy and control (FAC), family social support (FSS) and sense of belonging both at school (SSB) and in the neighbourhood (NSB). The largest differences were found for FAC (X^2 ^= 194.044, *p *= < 0.001, *V *= 0.244) and FAS (*X*^2 ^= 287.48, *p *= < 0.001, *V *= 0.282. For example, for FAC the distribution in England ranged from 69% to 3% compared to 46% and 10% for Spanish counterparts. Spanish respondents were much more likely to report that they lived in a family with low affluence (27.9%) compared with 6.6% in England).

Differences were also observed for FSS (,*X*^2 ^= 37.324. *p *= < 0.001, *V *= 0.108) SSB and NSB (*X*^2 ^= 130.558, *p *= < 0.001, *0056 *= 0.0.190; *X*^2 ^= 93.212, *p *= < 0.001, *V *= 0.168 respectively). In this regard Spanish respondents were more likely to report consistently higher levels across these attributes.

### Bivariate analysis

Tables 3 summarises the associations found between individual candidate assets and life satisfaction in each of the countries and all relationships were found to be significant. However, the magnitude of difference between countries was most marked for FAC (in the case of England, the effect size is much greater -Cohen's *d *= 1.34 than in Spain -Cohen's d = 0.82), which had a greater impact on life satisfaction in England. Mean values of life satisfaction in England range from 7.38 (high FAC) to 5.12 (low FAC) compared to the 7.51 (high FAC) to 6.23 (low FAC) in Spain.

Gender was significantly related to life satisfaction in both countries but family affluence only in England.

### General Linear Modelling (GLM)

Table 4 shows the results of the GLM and highlights that taken together the candidate assets included in the model explained 20% and 19% of the variance in life satisfaction in Spain and England respectively. For the purposes of this analysis candidate assets with partial eta squared estimates > 0.01 were considered to be important influences on life satisfaction.

Observing the models produced for both countries, 3 measures of social capital and 2 measures of social support were found to be important. However, the relative importance of these factors within each country differed. In Spain, FSS and SSB explained the largest amount of variance (F = 20.43 *p *< .0.001; partial η^2 ^= .031 and F = 18.33 *p *< .0.001; partial η^2 ^= .028 respectively). FAC and SSS explained less of the variance (1% each) but were still significant in the model. In England, whilst similar social capital and social support factors were shown to be important, a somewhat different configuration was observed. In this case, the candidate asset displaying the largest proportion of variance was FAC (F = 19.32, *p *< .0.001; partial η^2 ^= .027) followed by FSS and SSB (each explaining 3%) and then SSB (1%). Family affluence (FAS) was also highlighted as significant in England.

### Tree analysis

The decision trees (Figures 2 and 3) show how each of the independent variables interact to predict life satisfaction. All independent variables found to be significantly related to life satisfaction during bivariate analysis in the respective countries were included in this part of the analysis.

Each tree generated 3 levels, a different constellation of factors was observed for each country. In England, the optimum configuration of factors consisted of FAC (*p *= < 0.000, F = 36.272), SSB (*p *= < 0.000, F 29.442) and FSS (*p *= < 0.000, F 21.043). High levels of these potential assets reinforced the possibilities for improved life satisfaction from a base mean of 7.08 to 7.9. In contrast in Spain the maximum mean value of life satisfaction was achieved with high levels of FSS (*p *= < 0.000, F 61.329), SSS (*p *= < 0.000, F 30.466) and NSB (*p *= < 0.000, F 11.174), rising from 7.17 to 8.20.

Figures 2 and 3 also illustrate how decision trees can help to highlight assets that might be important when others at higher node levels are absent. For example, in Spain even those with medium levels of FSS (mean life satisfaction lower than mean for node 0), but have higher levels of school sense of belonging (SSS) can see observed improvements in life satisfaction.

## Discussion

Firstly, it is important to note that contrary to other reports [6] the study found the wellbeing of 15 year olds living in Spain and England to be similar, most reporting high levels of overall satisfaction with life. If pathways to health are to be articulated then this points to the need to work with precise definitions of health related concepts so that the antecedents and consequences of them can be determined.

There is some evidence amongst the sample population that social capital and social support can operate as protective factors and that it is possible to see an additive effect when more than one of them is in place. However, whilst a similar set of candidate assets were found to be important in both countries, their relative importance and interactions differed which may have implications for how initiatives are configured in Spain and England.

### Contexts

The findings concur with other studies [40], to suggest that even at age 15, the home and school remain important environments for building social capital.

#### Family

There is no shortage of evidence to demonstrate that building warm loving and positive relationships in the home are essential for securing the healthy development of children and young people [53]. One might have expected the older cohort of adolescents chosen for this study, to portray less reliance on the family for their wellbeing compared to other contexts. However, results from both the GLM and decision tree analysis showed that the family as a setting remained an important source of social capital and social support. In England, FAC (respondent's perception of how much say they had in making decisions about what they do) was shown consistently in both multivariate analyses to be protective of life satisfaction. The decision tree analysis showed that mean life satisfaction could be improved when high levels of FAC were observed. Duke et al. [54] have already shown that strong connections in the family can improve the likelihood of participation in more formal networks in adulthood. It may then be that families that provide open spaces for shared decision making are fostering the skills and competences required for active participation in community and civic life in the future.

In Spain, the candidate asset most commonly featured in both the GLM and the decision tree analysis was FSS (expressed as the ease of access to help and affection during times of difficulty) the latter highlighting significantly enhanced life satisfaction with high levels of FSS.

The intricacy of these findings suggests that the family as a unit needs to be understood in different country contexts. Fifteen year olds in England seem to put greater emphasis on becoming independent whereas Spanish adolescents gain more from direct family social support. These findings may reflect the nature of family life in general in Spain--albeit it changing-- that is orientations towards close bonds between family members and high levels of general support - often maintained into early adulthood [55]. That said, FSB, a sub component of social capital, characterised by the extent of families doing things together, did not appear in either multivariate analysis carried out for the Spanish sample.

#### School

The potential of school to be protective of health is well known [56]) and Morrow [40] found that from a young person's perspective, school was an important 'community' in its own right. In this study the role of school was found to be important but secondary to the impact that family life can have on wellbeing. In England SSB manifested through a range of positive relationships and feelings of security amongst classmates, is identified as another potential health asset. It features as a key predictor of life satisfaction in the GLM and a second level protective factor in the decision tree analysis. Those adolescents with both high levels of FAC and SSB achieve higher life satisfaction compared to the sample as a whole. Other school factors do not appear in either of the English multivariate analyses. In Spain, whilst SSB nears FSS in importance in the GLM, it is SSS (the feelings of social support available from the teacher), that feature in the decision tree as a second level protector of wellbeing. Life satisfaction continues to improve for those who have high levels of FSS and SSS. Spanish adolescents, as in the family context, seem to gain more from direct support provided by teachers than the feelings of belonging to a class group.

### Other contexts

It is well known that the peer group can contribute to the maintenance and reinforcement of both negative and positive health related outcomes, however in this study there was no evidence it was predictive of wellbeing in either country. This lack of evidence may have something to do with the definition of this particular candidate asset (a combination of same sex and opposite sex friendships). Stanton-Salazar and Spina [57] found friendships between adolescent males and females provided a more stable source of peer support, and therefore there may some masking of protective effect in our analysis. Other studies [58,59] have shown that neighbourhood social capital can be protective of a range of positive outcomes for young people. However, NSB was only marginally represented in the multivariate analysis results for Spain. Certainly, its absence from the English results may confirm Morrow's earlier work that young people to put greater importance on their interpersonal networks based on friendship and family to secure their sense of belonging and well-being. The absence of the neighbourhood indicator of social capital may be more to do with the fact that young people are often exclude from the possibilities to participate in community and civic life.

Finally, country differences were found in the relationship between FAS and life satisfaction. In England it was found to explain some of the variance in life satisfaction but to a lesser extent than the candidate assets associated with family and school. It was absent from all the analysis in Spain. This may be because whilst there may be a divide between those who are poorest in society and the rest of the population, the gap between those in the middle and higher strata is much less distinct.

### Country

At one level, this study clarifies that there is a consistency of relationship between the range of assets chosen for study and the life satisfaction of 15 year olds living in Spain and England. However on further examination, the study appears to affirm the idea that social capital is not a 'one size fits all' concept [24], and its optimum configuration needs to be understood alongside other related assets and specifically in relation to the cultural context within which it is being applied. The subtle differences found between Spain and England could reflect the broader cultural contexts between the 2 countries. At a general level, it is still true to say that societal values in Spain lean more towards the collective rather than the individual encouraging member's interdependence. This emphasizes the cultural and social relations associated with Mediterranean traditions especially in the role of the family as emotional support [60]. This might explain why in Spain, the social support assets have more prominence. Although, the reality is that in most Western societies, individualistic and collectivist cultural models act on a continuum, making the subtlety of these results difficult to interpret.

### Strengths and limitations

This study has a number of strengths. Firstly, HBSC survey provides a unique source of information on the social context of young people's lives allowing systematic examination of both the risks and protective factors that might determine health. It also, provides the ability to examine the precursors to health across a range of different cultures, hence testing the universality of social capital in different contexts. This study limited the comparisons to England and Spain to illustrate the impact that socio-cultural differences can have, however the HBSC provides the potential for the analysis to be replicated in a range of other country contexts.

Secondly, the health asset approach provides a helpful perspective by placing social capital as a positive resource during the development phase of adolescence and recognises that its individual constructs may be important as protective factors in their own right.

Finally it embraces the multi-dimensional nature of social capital to explore their interconnectedness compared to may other studies on social capital that have investigated single aspects. Whilst longitudinal studies are required to determine the linkages between its different indicators and importantly highlighting them as either antecedents or consequences of social capital, this study through its tree analysis has demonstrated how they might work together to maximise wellbeing.

As with all studies, there are a number of limitations. Most obviously, the cross sectional nature of HBSC limits the ability to deal with causation and therefore the direction of relationships found. That said, exploration of these relationships across different country contexts has helped to suggest some possible pathways to health for future investigation.

The composite indicators used to convey the sub domains of social capital and developed as part of the HBSC optional package have been validated for use in social capital research [23]. However, these indicators used existing items available in the study protocol and therefore further ongoing development is required. Whilst identified as a potential limitation, Earl and Carlson's [61] view is worthy of note, that is, evidence related to the social-environmental influences on child health and wellbeing can only be accrued if theory, measurement and analysis advance together. Building a complete and robust taxonomy of social capital indicators must therefore involve an iterative process of testing and re-development.

The family affluence scale used in this study has been found to be a good proxy indicator for measuring young people's socio-economic status [39], however the findings here would benefit corroboration through use of different measures of SES in future studies.

Some argue [26] that studies aiming to explore the links between social capital and health should use a multi-level approach to the analysis to take account of the different levels of influence. The findings from our individual level study of perceptions could be further enhanced by taking account of school and community level characteristics. These types of study would be a natural next step.

Due to data availability across both countries, it was not possible to explore the third dimension of social capital, social networking. This dimension seeks to assess the breadth and depth of young people's participation in a range of formal and informal networks. Other studies have shown this to be an important asset in its own right for various health related outcomes [23,62].

## Conclusions

There is some evidence to suggest that social capital (and its related concept of social support) do travel and are applicable in Spain and England. Some of its underlying constructs namely, family autonomy and control and school sense of belonging were found to be important predictors of wellbeing alongside more traditional indicators of social support. Given the different constellation of assets found in each country, it is not possible (or desirable) to define exactly the precise formula for applying social capital across cultures. This needs to be defined at the programme planning stage. Given the importance of family and school as settings for wellbeing, it is likely that individualistic notions of social capital are more prominent for young people whereby the social resources acquired through connections to others helps them to navigate their social environments. That said, the future potential for young people to contribute to social capital generation through participation in community life may depend on the accumulation of resources (health assets) accrued in theses contexts as they grow up.

## Competing interests

The authors declare that they have no competing interests.

## Authors' contributions

AM conceived and designed the study and made the first draft of the manuscript

FR performed the statistical analysis. CM participated in the design of the study and contributed to revisions to the manuscript. BJAH participated in redrafts of the manuscript. All authors read and approved the final manuscript

## Pre-publication history

The pre-publication history for this paper can be accessed here:

http://www.biomedcentral.com/1471-2458/12/138/prepub
